# Scaling of Haralick features with image bit depth and gray level co-occurrence matrix displacement vector for linear gradients

**DOI:** 10.1016/j.compbiomed.2025.110880

**Published:** 2025-08-13

**Authors:** Ana Oprisan, Sorinel Adrian Oprisan

**Affiliations:** Department of Physics and Astronomy, College of Charleston, Charleston, SC 29424, USA

**Keywords:** Linear-gradient pattern, Estimated Haralick feature, Gray level quantization, Displacement vector

## Abstract

**Background and Objective:**

Perceptual studies have shown that textures that are indistinguishable based on their second-order statistics are perceived as equivalent by the human visual system. These statistics, which capture spatial correlations in pixel intensities, are more closely related to image gradients than to absolute pixel values. This study aims to derive analytic scaling laws for Haralick texture features to enable quantization-invariant, reproducible texture classification across heterogeneous imaging conditions.

**Methods:**

We analyzed the symmetries of the Gray-Level Co-occurrence Matrix (GLCM) induced by linear image gradients of ∇ gray levels per pixel. Exploiting these structural regularities, we derived closed-form scaling laws for four widely used Haralick features—Energy, Contrast, Correlation, and Inverse Difference Moment. These laws yield theoretically justified normalization factors that reduce or eliminate dependence on image bit depth and gray-level quantization $\left(N_{g}\right)$.

**Results:**

Linear gradients produce GLCM entries aligned along diagonals offset by $\nabla \cdot \left|d\right|$, where d is the displacement vector. This structure enabled the derivation of analytic expressions describing each feature’s scaling behavior, validated through numerical simulations on synthetic images. The derived normalization factors outperform prior empirical approaches and explain observed discrepancies in real-world datasets.

**Conclusions:**

Our results provide a principled framework for the normalization of Haralick features, improving reproducibility, comparability, and interpretability across heterogeneous datasets. The scaling laws also inform feature selection and classifier design by identifying which features are most robust to quantization and displacement. These insights enable efficient and standardized texture analysis for applications in radiomics, remote sensing, and computer vision.

## 1. Introduction

### Biomedical applications of texture classification.

Medical imaging is a critical component of modern healthcare, employed for disease detection, treatment planning, and prognosis prediction. Legal and ethical standards mandate that a licensed physician make or confirm the final diagnosis based on medical images [1]. The growing dependence on imaging for diagnostic purposes has led to a substantial increase in the number of images requiring analysis and classification by human clinicians. Furthermore, in many countries, the double-reading of medical images is required to ensure diagnostic accuracy. Given increasingly stringent screening guidelines and a limited pool of qualified clinicians, the workload of clinical radiologists has risen significantly in recent years [2]. Among other quantitative measures, it is noteworthy that “teaching radiologists’ aggregate total workload showed a cumulative growth of 84% from 2008 to 2019” [3].

Recent advances in machine learning (ML) and artificial intelligence (AI) have made medical imaging the most prominent domain for AI applications. AI-based pre-screening of medical images can reduce the volume of data by 61.72%, eliminating cases that do not require human review [1]. Replacing the second reader in clinical workflows with AI has reduced the number of images reviewed by human clinicians by 44.47% [1]. As of 2025, more than 340 AI tools have been approved by the U.S. Food and Drug Administration for clinical use in radiology. AI systems are now integrated into the daily operations of approximately two-thirds of radiology departments in the United States. In addition to alleviating clinician workload, ML and AI algorithms can also leverage the approximately 97% of currently unused hospital data to support predictive treatment [4,5].

### Motivation and contribution.

The human visual system accurately identifies and classifies textures in biomedical images by naturally computing second-order statistics. To achieve comparable performance, ML and AI algorithms also leverage second-order statistics—most notably through the use of gray-level co-occurrence matrices (GLCMs) and Haralick features for texture classification. However, a key gap remains in aligning these computational descriptors with human visual perception: the features are sensitive to image bit depth, which hinders cross-dataset comparisons and limits the portability of results. Only a few studies have explored Haralick feature normalization for cross-modality comparisons [6,7], and even recent methods leave many features dependent on image quantization settings [8]. This disconnect undermines the interpretability and generalizability of findings across imaging modalities and acquisition protocols. Further compounding this issue is the sensitivity of Haralick features to quantization parameters such as the number of gray levels $\left(N_{g}\right)$ and the specific quantization method used. These dependencies severely constrain reproducibility, comparability, and clinical deployment.

### Second-order image statistics and Haralick features.

A critical component of medical image analysis is texture classification, which has traditionally relied on human vision. Humans are highly effective at identifying textures due to the visual system’s sensitivity to local contrast and luminance gradients rather than absolute intensities [9,10]. These gradients are captured by second-order statistics, such as the joint distribution of gray-level pairs separated by a given distance [11,12]. This biological insight has inspired algorithmic approaches for medical image segmentation and classification [13].

The joint distribution of gray-level pairs i and j, separated by a displacement vector d, defines the GLCM [14,15]. It captures image gradients that are naturally processed by human vision. GLCM values encode the relative magnitude of intensity change $\left|i-j\right|$ across a distance d, which serve as the basis for Haralick’s 14 statistical texture features [16,17]. These features have been widely adopted in biomedical imaging due to their discriminative power and robustness to noise [18], with applications spanning X-ray [19,20], CT [21], PET [22], ultrasound [23], and dermatology [24].

### Quantization sensitivity and reproducibility limitations.

A major limitation of Haralick features is their dependence on the number of gray levels $N_{g}$. For example, an 8-bit image yields a 256 × 256 GLCM, while a binary image produces a 2 × 2 GLCM. This variability introduces significant challenges when comparing datasets acquired with different bit depths or quantization schemes [25–27]. Further complicating the issue, the optimal bit depth depends on image size, the signal-to-noise ratio (SNR) [28,29], and content [30]. Consequently, the sensitivity of Haralick features impedes standardization across imaging protocols and clinical centers.

### Gradient models and GLCM symmetries.

To address these limitations, we derive analytic scaling laws that describe how Haralick features depend on bit depth, gradient strength, and pixel displacement. We simplify the problem by focusing on one-dimensional gradients in two-dimensional images, revealing symmetry properties of the GLCM. This simplification is justified by the fact that many real-world texture features — such as color and structure — are gradient-based and exhibit statistical invariance [31–33]. Each pixel encodes a local gradient component, whose histogram forms the gradient distribution. We demonstrate how gradient-induced GLCM symmetries yield closed-form relationships for Haralick energy, contrast, correlation, and homogeneity.

### Comparison with other gradient-based methods.

Alternative gradient-based approaches include Histogram of Oriented Gradients (HOG) [34], Scale-Invariant Feature Transform (SIFT) [35], and Gradient Local Auto-Correlations (GLAC) [36]. GLAC extend gradient analysis to second-order statistics and capture both spatial and orientational correlations. Other techniques include local binary patterns [37], wavelets [38], and fractal dimensions [39]. While Haralick features are simpler, they offer lower computational cost and remain attractive for clinical applications. Haralick features have also been combined with 3D structure tensor methods in diffusion MRI to infer brain connectivity [40].

### Scope and contributions.

To our knowledge, this is the first study to derive closed-form scaling laws for Haralick features as functions of gradient magnitude ∇, gray-level count $N_{g}$, and pixel displacement d. These laws yield normalization factors that make Haralick features asymptotically invariant to quantization settings. We validate our analytic expressions using synthetic linear-gradient images. Our contributions are as follows:

1. Identification of GLCM symmetries induced by linear gradients (Section 3.1);
2. Derivation of analytic scaling laws for four Haralick features (Section 3.2);
3. Simulation-based validation of these laws (Section 3.2);
4. Consistent identification of normalization factors for bit-depth invariance (Section 3.2).

### Broader implications.

Although developed for biomedical imaging, our scaling laws have broader applicability. In remote sensing, Haralick features are used for terrain classification [41]. In industrial quality control, they are used to detect defects under varying imaging conditions. In digital pathology, they enhance robustness to staining and scanner variability [42, 43]. Our analytic framework enables reproducible, device-independent texture analysis across disciplines.

### Selecting the most representative Haralick features.

There are several reasons why we focused this study on the four Haralick features: Energy, Contrast, Correlation, and Inverse Difference Moment (IDM). Several of the 14 Haralick features are mathematically correlated or describe similar statistical properties. In particular, Sum Variance and Sum Average are highly correlated with Contrast. Another example is Difference Variance, which often overlaps in behavior with Variance or Contrast. Including such correlated features adds dimensionality without improving performance and can worsen generalization.

Another reason we considered only these four features is that some of the others exhibit low robustness to noise and quantization. Features like Maximum Probability, Sum Entropy, and Difference Entropy tend to be highly sensitive to quantization artifacts and local texture noise. In real-world images — especially those affected by compression, digitization, or biomedical acquisition — these features fluctuate more than stable features like Energy or IDM.

A further consideration is computational and analytical complexity. Some features, such as the Information Measures of Correlation 1 and 2, involve joint entropy terms and logarithmic expressions with potential division by small values, making them highly susceptible to rounding and floating-point instability. This complexity makes them harder to interpret analytically and more difficult to model through scaling laws.

Some features also lack discriminative power. Empirical studies have shown that a small subset of features — often just three to five — captures the majority of discriminative power for many classification or regression tasks. The rarely used features contribute marginally, if at all, once the main features are included.

Finally, some of the more complex Haralick features exhibit subtle differences depending on the implementation (e.g., different entropy or normalization strategies). This variability creates inconsistencies across libraries and studies, reducing their reliability in comparative pipelines.

### Organization of the paper.

Section 2.1 reviews the GLCM framework. Appendix A defines the four selected Haralick features used in this study. Section 3.1 introduces new GLCM symmetries that arise in linear gradient images. Building on these symmetries, Section 3.2 derives analytic scaling laws for each of the four selected Haralick features. The corresponding scaling laws are numerically validated using synthetic one-dimensional linear gradient images. These scaling laws are then used to derive normalization factors that render Haralick features invariant to image bit depth. Section 4 examines the dependence of Haralick features on image quantization schemes, as reported in the literature—most notably in [8,44]. These studies, based on real-world biomedical datasets, confirm all of our scaling law predictions. They also provide a foundation for comparing our proposed normalization factors against previously suggested empirical alternatives. A brief comment on MATLAB and Python implementations of all Haralick features is presented in Appendix B.

## 2. Methods

### 2.1. Gray-Level Co-occurrence Matrix (GLCM)

This section reviews the procedure for mapping image (pixel) space $N_{x}\times N_{y}$ to the $N_{g}\times N_{g}$ space of the joint probability distribution represented by the GLCM. This provides the foundation for our novel results on GLCM symmetry induced by image gradients, presented in Section 3.1.

**Fig. 1.**
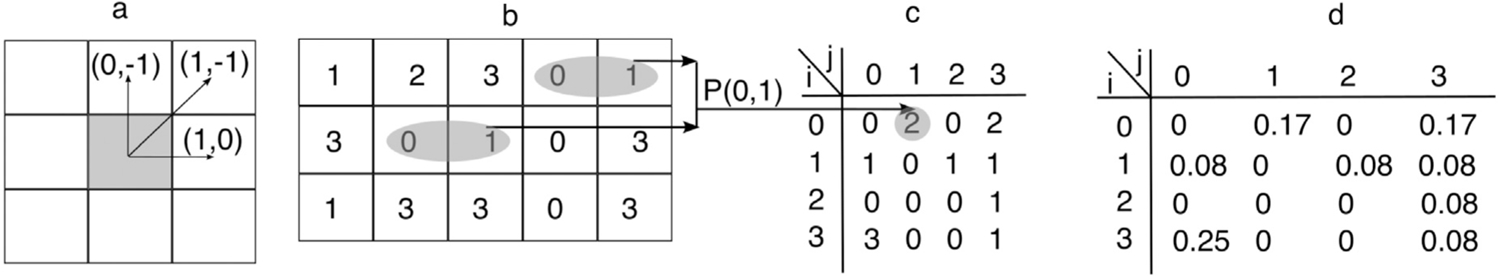
Gray-Level Co-occurrence Matrix (GLCM). (a) Neighbors of the central pixel (shaded area) are identified by the displacement (offset) vector $d=\left(\Delta x,\Delta y\right)$. For example, the first-order upward-right neighbor of the central pixel has a displacement vector $d=\left(\Delta x=1,\Delta y=-1\right)$. (b) A 5 × 3 image with 2-bit depth. For a horizontal displacement vector $d=\left(1,0\right)$, the two elliptical shaded areas indicate the only two pixel pairs with gray-level intensities i=0 and j=1. (c) The GLCM $P_{d}\left(i,j\right)$ of the image from panel (b) for $d=\left(1,0\right)$. The shaded GLCM entry $P_{d}\left(i=0,j=1\right)=2$ corresponds to the two elliptical pixel pairs with intensities i=0 and j=1, separated by displacement $d=\left(\Delta x=1,\Delta y=0\right)$ (see panel (b)). (d) The corresponding normalized GLCM $p_{d}\left(i,j\right)$.

The light intensities in a two-dimensional $N_{x}\times N_{y}$ grayscale image $I\left(x,y\right)$ are quantized and stored as an array of positive integers with $N_{g}$ gray levels. A Cartesian reference frame is anchored at the upper-left corner of the image, as shown in Fig. 1a. The displacement vector $d=\left(\Delta x,\Delta y\right)$ specifies the location of a target pixel with intensity j and coordinates $\left(x_{j},y_{j}\right)$ relative to a reference pixel with intensity i and coordinates $\left(x_{i},y_{i}\right)$. Here, $\Delta x=x_{j}-x_{i}$ represents the rightward (horizontal) displacement, and $\Delta y=y_{j}-y_{i}$ represents the downward (vertical) displacement.

The example in Fig. 1b shows the light intensities of an artificial $N_{x}\left(=5\right)\times N_{y}\left(=3\right)$ image encoded with a bit depth b=2, such that $N_{g}=2^{b}=4$ gray levels.

The GLCM counts the number of co-occurrences between a reference gray-level i and a target gray-level j, separated by a displacement vector $d=\left(\Delta x,\Delta y\right)$ [16]:

(1) $$P_{d}\left(i,j\right)=\#\left\{\left(\left(x_{i},y_{i}\right),\left(x_{j},y_{j}\right)\right):I\left(x_{i},y_{i}\right)=i\;\&\;I\left(x_{j},y_{j}\right)=j\right\},$$

where # denotes the cardinality of the set. The coordinates of the reference pixel with gray level i are $\left(x_{i},y_{i}\right)$, and the coordinates of the target pixel with gray level j are $\left(x_{j}=x_{i}+\Delta x,y_{j}=y_{i}+\Delta y\right)$.

For example, for a horizontal unit displacement vector $d=\left(\Delta x=1,\Delta y=0\right)$, the GLCM of the image shown in Fig. 1b is depicted in Fig. 1c. In Fig. 1b, there are only two shaded pixel pairs where i=0 and j=1 at displacement $d=\left(1,0\right)$, which correspond to the GLCM entry $P_{d}\left(0,1\right)=2$ in Fig. 1c.

In practice, the normalized GLCM $p_{d}\left(i,j\right)$ is typically used. It represents the probability of observing gray level i at a displacement d from a pixel with gray level j [16,17]:

(2) $$p_{d}\left(i,j\right)=\frac{P_{d}\left(i,j\right)}{\sum_{i=0}^{N_{g}-1}\sum_{j=0}^{N_{g}-1}P_{d}\left(i,j\right)}.$$

As an example, in an $N_{x}\times N_{y}$ image, the total number of horizontal and vertical pixel pairs at unit distance $\left|d\right|=1$ are given by:

$$R_{x}=\sum_{i=0}^{N_{g}-1}\sum_{j=0}^{N_{g}-1}P_{\left(1,0\right)}\left(i,j\right)=\left(N_{x}-1\right)N_{y},$$

$$R_{y}=\sum_{i=0}^{N_{g}-1}\sum_{j=0}^{N_{g}-1}P_{\left(1,0\right)}\left(i,j\right)=\left(N_{y}-1\right)N_{x}.$$

Therefore, for the image in Fig. 1b with $N_{x}=5$ and $N_{y}=3$, the normalization factors are $R_{x}=12$ and $R_{y}=10$, respectively. The corresponding normalized GLCM is shown in Fig. 1d. For instance, $p\left(0,1\right)=\frac{P\left(0,1\right)}{R_{x}}=\frac{2}{12}\approx 0.17$.

While the GLCM provides a significant dimensionality reduction from the image space $N_{x}\times N_{y}$ to the gray-level space $N_{g}\times N_{g}$, Haralick features further reduce the dimensionality of the bivariate distribution $p_{d}\left(i,j\right)$ to a set of scalar values, as defined in Appendix A.

We emphasize that the definitions of Haralick features (see Appendix A and [16,17]) do not specify how these features scale with key image properties, such as the number of gray levels $N_{g}$, the displacement vector $d=\left(\Delta x,\Delta y\right)$, or the image gradient magnitude ∇. Here, ∇ refers to the average rate of change in intensity across neighboring pixels, typically computed using finite differences or directional derivatives.

For example, the definition of Haralick Energy in Eq. (16) specifies only how to compute its value from the entries of the GLCM. The definition does not indicate whether Energy scales linearly, quadratically, or otherwise with the number of gray levels $N_{g}$. Similarly, the definition of the Haralick feature Contrast $f_{2}$ (Eq. (17)) offers no analytic guidance on how it varies with parameters such as the pixel displacement vector d.

Understanding the scaling behavior of Haralick features with respect to the number of gray levels $N_{g}$, the image gradient magnitude ∇, and the displacement vector d is critical for comparing images across modalities, resolutions, or acquisition protocols [6,7]. Despite their widespread empirical use, little theoretical work has addressed this issue.

This study presents a coherent framework for deriving closed-form scaling laws for Haralick features. These scaling laws enable the identification of redundancies among features, the quantification of sensitivity to image parameters, and the introduction of normalization factors that render Haralick features asymptotically independent—with respect to image bit depth, for example. This analytical approach provides a theoretical foundation for improving reproducibility and comparability in texture-based image analysis.

## 3. Results

### 3.1. GLCM symmetry induced by linear gradients

Although the GLCM was introduced over five decades ago, the relationship between image gradients and the resulting GLCM symmetries has been neither thoroughly explored nor leveraged for the analytic derivation of Haralick features. We utilize the observation that natural scene gradients exhibit weak negative correlations between orthogonal components [45], allowing us to treat them as statistically independent. To further simplify the analytic derivations, this study focuses exclusively on one-dimensional gradients.

**Fig. 2.**
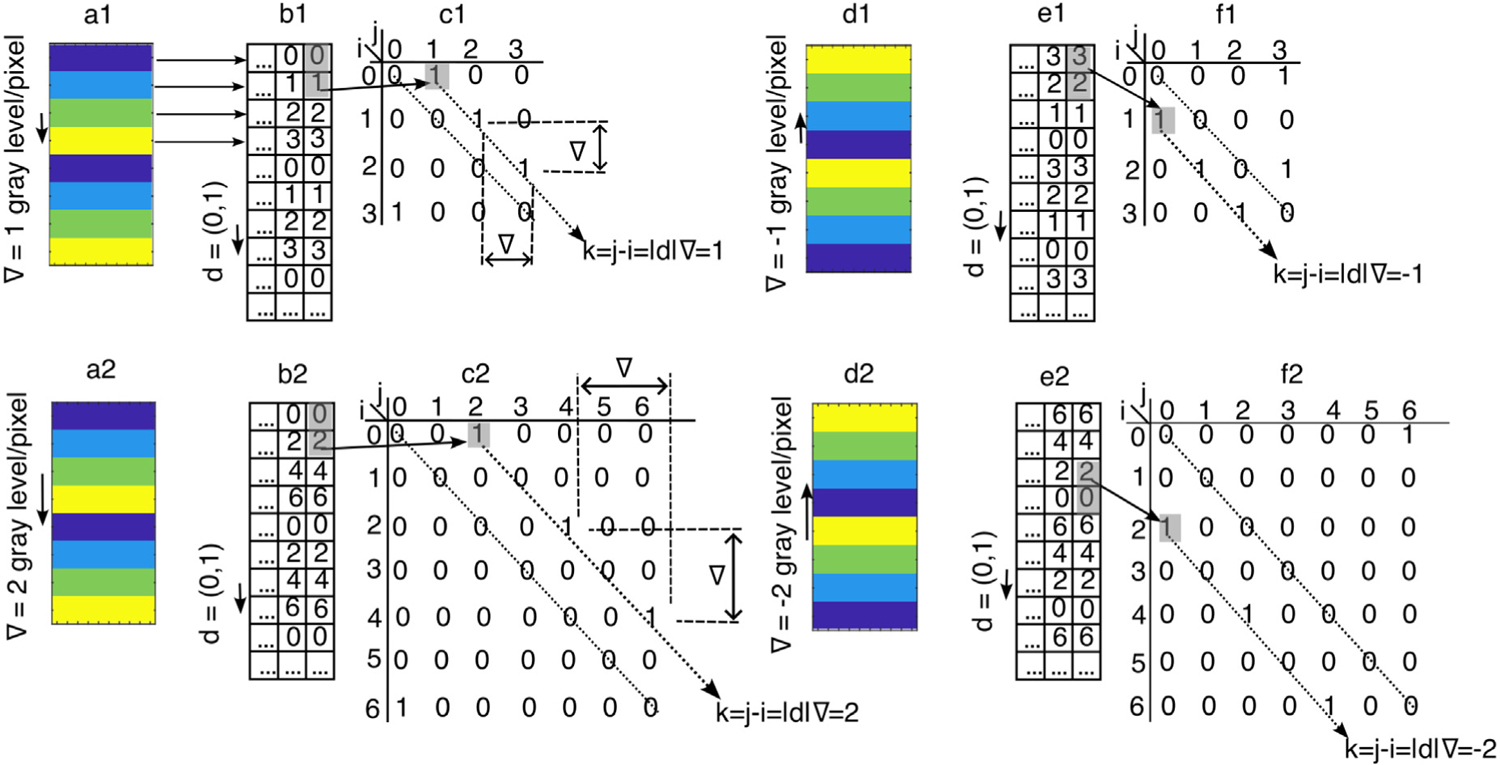
Symmetries in the GLCM $P_{d}\left(i,j\right)$ induced by linear vertical gradients. (a1) Horizontal stripes with vertical gradient $\nabla_{y}=1$, color-coded. (b1) Quantized gray levels from panel (a1). (c1) GLCM $P_{d}\left(i,j\right)$ corresponding to (b1). (d1–f1) Descending gradient $\nabla_{y}=-1$. (d2–f2) Gradient $\nabla_{y}=-2$. GLCMs correspond to one period of the pattern.

A one-dimensional linear gradient in an image (see Fig. 2a1) induces symmetries in the corresponding GLCM, which we exploit to derive analytic expressions for the scaling behavior of Haralick features. Here, ∇ denotes the image gradient, defined as the number of gray-level steps per unit pixel displacement in the direction of interest.

For example, Fig. 2a1 shows a color-coded 2-bit $\left(b=2\right)$ bit-depth image with a vertically increasing (positive) gradient of ∇=1 gray level per pixel. The gradient magnitudes are indicated along the left side of each panel in Fig. 2. Specifically, panel a1 has ∇=1, panel a2 shows ∇=2, panel d1 has ∇=−1, and panel d2 shows ∇=−2. Arrows along the left edge of the respective panels indicate the gradient direction.

Horizontal arrows from Fig. 2a1 to b1 illustrate the mapping from grayscale intensities to quantized values. For instance, the first row of pixels in Fig. 2a1 is mapped to a row of zeros in Fig. 2b1. Vertical arrows in panels b1, b2, e1, and e2 indicate the direction of the vertical displacement vector $d=\left(0,1\right)$.

Our main results regarding GLCM symmetries for images with $N_{g}$ gray levels and linear gradients are summarized below:

1. **The first nonzero GLCM entry is $P_{d}\left(i=0,j=\left|d\right|\nabla \right)$.** For example, in Fig. 2c1 with ∇=1, the first nonzero GLCM entry is at i=0 and j=1. In Fig. 2c2 with ∇=2, it occurs at j=2. This remains true even when ∇=1 is sampled with $d=\left(0,2\right)$, since $j=\left|d\right|\nabla =2$.
2. **Nonzero GLCM entries are spaced horizontally and vertically by ∇ gray levels.** The vertical gradients in Fig. 2 have a 1-pixel resolution, so Δi=Δj=∇. These offsets are independent of $\left|d\right|$ (see Fig. 2c1, c2, f1, and f2).
3. **Only GLCM entries along $k=j-i=\left|d\right|\nabla$ are nonzero for positive gradients.** These entries lie parallel to and above the GLCM’s principal diagonal. For negative gradients, the nonzero entries lie parallel to and below the principal diagonal.

The GLCM symmetries described above are general and apply to any image with a one-dimensional linear gradient. However, these symmetries depend on both the number of gray levels $N_{g}$ and the displacement vector d used to probe the image texture. We further observe that:

1. Each nonzero GLCM entry has a reference pixel intensity i from the set $\left\{0,\nabla ,2\nabla ,\ldots ,\left({\tilde{N}}_{g}-1\right)\nabla \right\}$, where
(3) $${\tilde{N}}_{g}=1+\left\lfloor \frac{N_{g}-1}{\left|\nabla \right|}\right\rfloor ,$$
  is the number of nonzero GLCM entries. For example, for $N_{g}=7$ and ∇=2 (Fig. 2a2), we have ${\tilde{N}}_{g}=1+\left\lfloor \left(7-1\right)/2\right\rfloor =4$.
2. The total number ${\tilde{N}}_{g}$ of nonzero entries is independent of $\left|d\right|$. As shown in Eq. (3), it depends only on $N_{g}$ and ∇.

Fig. 2, Panels d1–f1 and d2–f2, illustrate the negative gradient cases for ∇=−1 and ∇=−2, respectively.

**Fig. 3.**
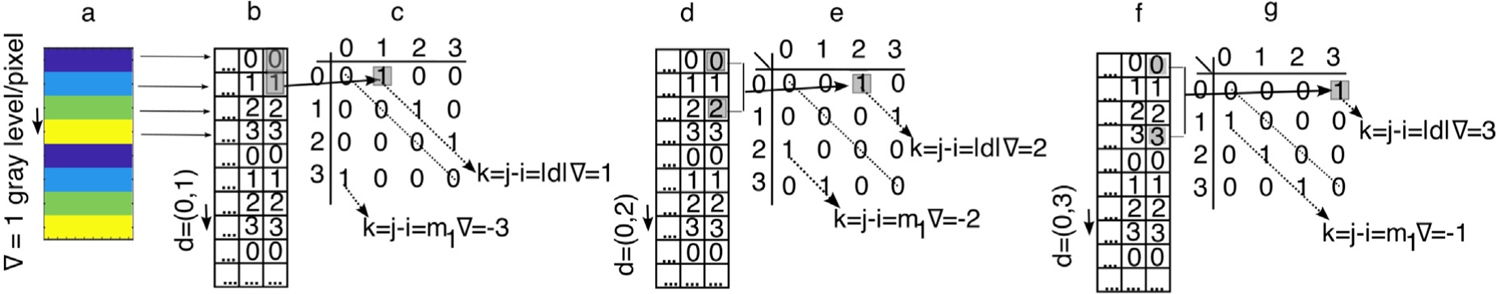
GLCM for different displacement vectors. (a) Horizontal stripes with a vertical gradient $\nabla_{y}=1$ in a b=2-bit depth image. (b) Quantized gray levels. (c) GLCM for $d=\left(0,1\right)$. (d–e) GLCMs for $d=\left(0,2\right)$ and $d=\left(0,3\right)$, illustrating $m_{1}$ and $m_{2}$.

The displacement vector d also plays a significant role in GLCM symmetry. We find that d determines both the position and magnitude of nonzero GLCM entries, as well as the scaling behavior of Haralick features. As demonstrated in Section 3.2 and illustrated in Figs. 2 and 3, changes in d shift the distribution of entries relative to the GLCM’s principal diagonal. Additionally, both $N_{g}$ and d jointly influence the symmetry structure of the GLCM:

1. **The offset $\left|d\right|\nabla$ for $P_{d}\left(0,\left|d\right|\nabla \right)$ defines $m_{1}$, the number of entries above the GLCM’s principal diagonal.** If $\left|d\right|=1$, then $m_{1}\approx \left(N_{g}-1\right)/\nabla$. However, when $j>N_{g}-1$, entries wrap around due to periodic boundary conditions in gray-level space:
(4) $$m_{1}={\tilde{N}}_{g}-\left|d\right|\nabla .$$
  In Fig. 3c, with $d=\left(0,1\right)$, ∇=1, and $N_{g}=4$, we have ${\tilde{N}}_{g}=4$, so $m_{1}=3$. Entries range from $P_{d}\left(0,1\right)$ to $P_{d}\left(2,3\right)$.
2. **Entries exceeding $N_{g}-1$ wrap around modulo $N_{g}$.** These wrapped entries lie parallel to and below the GLCM’s principal diagonal, starting from $P_{d}\left(m_{1}\nabla ,0\right)$, $P_{d}\left(\left(m_{1}+1\right)\nabla ,\nabla \right)$, and so on.
3. The number of entries parallel to and below the GLCM’s principal diagonal is given by:
(5) $$m_{2}={\tilde{N}}_{g}-m_{1}=\left|d\right|\nabla .$$
4. **All $m_{2}=\left|d\right|\nabla$ entries parallel to and below the GLCM’s principal diagonal lie along $k=j-i=-m_{1}\nabla$**. This symmetry arises from the periodic wraparound at $N_{g}-1\to 0$, which partitions the set of ${\tilde{N}}_{g}$ entries into two subsets along $k=\pm \left|d\right|\nabla$.

### 3.2. Scaling laws for Haralick features with linear gradients

It is intuitive that the number of gray levels $N_{g}$ directly influences texture measures such as Haralick features. Fewer gray levels result in the loss of fine texture details, while more gray levels increase GLCM sparsity and sensitivity to noise. Different quantization schemes can significantly alter Haralick feature values, making cross-study or cross-dataset comparisons unreliable without appropriate normalization. Variations in lighting conditions or imaging resolution also affect image gradients, which renders Haralick features sensitive to factors unrelated to intrinsic image content. This parameter sensitivity has been previously documented (see [18] and references therein), and it complicates the standardization of texture classification in the absence of consistent and well-justified normalization strategies.

Our goal is to utilize GLCM symmetries to analytically derive scaling laws obeyed by Haralick features, thereby providing a consistent foundation for defining feature normalization factors. While the most common image acquisition parameter affecting image quality and Haralick features is the number of gray levels $N_{g}$, we are also interested in how these features scale with the image gradient ∇ and the displacement vector d. We justify our selection of normalization factors based on analytically derived scaling laws.

For example, assume a Haralick feature f scales with $N_{g}$ according to a power law with exponent α, i.e., $f\propto N_{g}^{\alpha }$. To compensate for its divergence as $N_{g}$ increases and to make it independent of the number of gray levels, one multiplies it by $N_{g}^{-\alpha }$, yielding the normalized Haralick feature $\tilde{f}=fN_{g}^{-\alpha }$. Consistent and theoretically justified normalization factors that render Haralick features invariant to the quantization level $N_{g}$ are critical for comparing features across different quantization schemes and for improving reproducibility [6,7,18,25,26,28,30].

The results presented in Section 3.1 simplify the derivation of scaling laws for Haralick features [16,17]. These scaling laws can be used, for example, to estimate the strength of image gradients based on the values of Haralick features.

Each of the following subsections focuses on one of the four selected Haralick features and is organized as follows:

- Systematic derivation of the Haralick feature scaling laws.
- Comparison of analytic predictions against numerically computed Haralick features obtained from synthetic images with one-dimensional linear gradients.
- Determination of normalization factors from the scaling laws and comparison with empirical approaches reported in the literature.

**Fig. 4.**
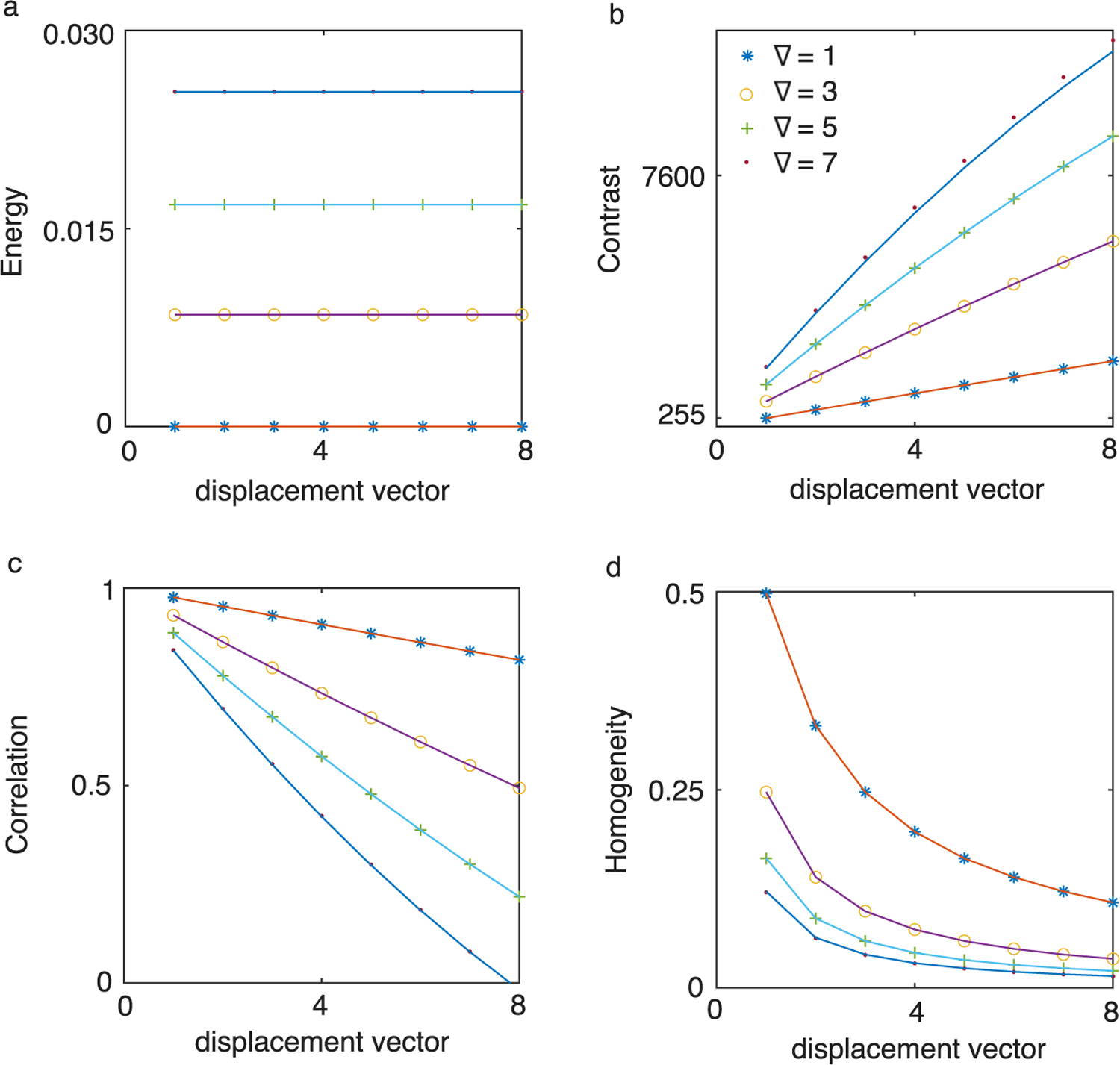
Analytical vs. numerically calculated features. All features were computed for vertical displacements $\left|d\right|=1,\ldots ,8$ and gradients $\nabla =1\left(“*”\right)$, 3 (“o”), 5 (“+”), and 7 (“.”). The gradient images have 1024 × 1024 pixels and 8-bit depth. (a) ASM $\left(f_{1}\right)$ is independent of $\left|d\right|$ and matches Eq. (6). (b) CON $\left(f_{2}\right)$ increases with ${\left|d\right|}^{2}$, as predicted analytically by Eq. (9). (c) COR $\left(f_{3}\right)$ also shows ${\left|d\right|}^{2}$ dependence, consistent with Eq. (12). (d) IDM $\left(f_{5}\right)$ agrees with the prediction from Eq. (23).

We generated 1024 × 1024-pixel, 8-bit synthetic images with varying vertical gradient intensities ∇. GLCMs were computed using the MATLAB function graycomatrix(), and Haralick features were calculated using graycoprops(). All features were evaluated over a range of gradient intensities $\nabla =\left\{1,\ldots ,7\right\}$ gray levels per pixel and vertical displacement vectors $d=\left\{1,\ldots ,8\right\}$, as shown in Fig. 4.

#### 3.2.1. Scaling law for Angular Second Moment (ASM) or energy

**Energy**, also known as the **Angular Second Moment (ASM)**, is defined in Eq. (16). Its computation requires only the normalized GLCM values $p_{d}\left(i,j\right)$. The Energy feature measures image uniformity.

High ASM values indicate low texture complexity, homogeneous regions, and the dominance of specific pixel pairs, such as those found in structured or repetitive patterns. Low ASM values reflect high texture complexity, with varied or chaotic image regions often associated with noise or rough surfaces. The ASM values are bounded: $f_{1}\in \left[0,1\right]$. ASM approaches 1 when the GLCM contains a single dominant entry. Indeed, for a uniform image with intensity i, there is only one nonzero GLCM entry, $p_{d}\left(i,i\right)=1$, which yields $f_{1,\max}=1$. ASM decreases as the co-occurrence distribution becomes more dispersed. In the limiting case of a random image where all ${\tilde{N}}_{g}$ GLCM entries are equal to $1/{\tilde{N}}_{g}$, the result is $f_{1,\min}=1/{\tilde{N}}_{g}$. Thus, for any image, the following tighter bounds hold:

$$f_{1}\in \left[\frac{1}{{\tilde{N}}_{g}},1\right].$$

*3.2.1.1.* For one period of a linear gradient, with ∇ gray levels per pixel, all ${\tilde{N}}_{g}$ nonzero entries of the GLCM $P_{d}\left(i,j\right)$ defined in Eq. (3) are equal to one (see Figs. 2 and 3). Since these GLCM entries repeat uniformly, their normalized values are equal to $p_{d}\left(i,j\right)=1/{\tilde{N}}_{g}$. Therefore, the ASM feature becomes:

(6) $$f_{1}=\sum_{i=0}^{N_{g}-1}\sum_{j=0}^{N_{g}-1}p_{d}{\left(i,j\right)}^{2}=\frac{1}{{\tilde{N}}_{g}^{2}}\cdot {\tilde{N}}_{g}=\frac{1}{{\tilde{N}}_{g}}=\frac{1}{1+\left\lfloor \frac{N_{g}-1}{\left|\nabla \right|}\right\rfloor }.$$

Note that the analytical expression for $f_{1}$ is independent of the displacement vector d for linear gradients. This is because d merely shifts the positions of GLCM entries relative to the principal diagonal without altering their magnitudes. According to Eq. (6), $f_{1}$ decreases with increasing $N_{g}$ and increases with ∇, since a larger ∇ results in fewer nonzero GLCM entries and higher individual values of $p_{d}\left(i,j\right)$ [46]. We summarize the scaling behavior as $f_{1}\left(N_{g},\nabla \right)\propto \nabla^{+1}N_{g}^{-1}$.

Real-world results for benign glandular structures, shown in the lower-left panel of Fig. 5 from [8], exhibit an exponential decrease in the Haralick feature $f_{1}$ with $N_{g}\in \left\{32,64,96,128,160,192,224,256\right\}$, consistent with our theoretical prediction from Eq. (6).

*3.2.1.2.* Representative numerical results for $f_{1}$ with gradient values $\nabla =\left\{1,3,5,7\right\}$ are shown in Fig. 4a. As predicted by Eq. (6), $f_{1}$ is independent of the displacement vector d. The values of $f_{1}$ for different gradients are vertically spaced according to the analytical prediction in Eq. (6).

*3.2.1.3.* Löfstedt et al. proposed an empirical normalization of the Haralick feature $f_{1}$ (see Eq. (16)) as $f_{1,\text{Lofstedt}}=f_{1}N_{g}^{2}$. However, our analytical result (Eq. (6)) contradicts this empirical factor, showing that $f_{1}$ decreases linearly with $N_{g}$, not quadratically. Real-world results for benign glandular structures, shown in the lower-right panel of Fig. 5 from [8], indicate that after their empirical normalization, $f_{1,\text{Lofstedt}}$ increases (nonlinearly) with $N_{g}$ instead of becoming independent of it, as expected. This change in the convexity of $f_{1}$ after empirical normalization indicates that the $N_{g}^{2}$ factor overcompensates.

Clausi, [44] also reported reduced classification performance at high $N_{g}$. Our result in Eq. (6) supports this finding, as $f_{1}$ decreases inversely with the number of gray levels, reducing its discriminative power. Therefore, the correct normalization to make $f_{1}$ invariant to $N_{g}$ is:

(7) $${\tilde{f}}_{1}=f_{1}N_{g}=N_{g}\sum_{i=0}^{N_{g}-1}\sum_{j=0}^{N_{g}-1}p_{d}{\left(i,j\right)}^{2}.$$

#### 3.2.2. Scaling law for contrast (CON)

**Contrast (CON)**, defined in Eq. (17), is computed using the marginal distribution $p_{x-y}\left(k\right)$, which aggregates all GLCM elements with a fixed offset $k=\left|i-j\right|$ (see Eq. (18) in Appendix A). The Haralick Contrast feature quantifies local intensity variation in an image based on the difference in gray levels between neighboring pixel pairs. High Haralick Contrast indicates significant intensity variation, typically observed in edge-rich, rough, or highly textured regions, which results from the prevalence of pixel pairs with large gray-level differences. Low Haralick Contrast, by contrast, characterizes uniform or smooth regions with minor intensity variation, suggesting relatively flat or consistent textures. Haralick Contrast values are non-negative and typically unbounded above, depending on the gray-level range $N_{g}$ of the image. The minimum value of Contrast is 0, which occurs when all pixels have the same gray level.

*3.2.2.1.* Using the GLCM symmetries derived in Section 3.1, the marginal probability $p_{x-y}\left(k\right)$ is nonzero only for GLCM entries parallel to the principal diagonal:

(8) $$k=j-i=\left\{\begin{array}{ll} \left|d\right|\nabla , & \begin{array}{l} \text{for}\;\text{the}\;m_{1}={\tilde{N}}_{g}-\left|d\right|\nabla \;\text{entries}\;\text{above}\\ \text{the}\;\text{GLCM's}\;\text{principal}\;\text{diagonal}\;\left(\text{Eq}.\left(4\right)\right), \end{array}\\ -m_{1}\nabla , & \begin{array}{l} \text{for}\;\text{the}\;m_{2}=\left|d\right|\nabla \;\text{entries}\;\text{below}\\ \text{the}\;\text{GLCM's}\;\text{principal}\;\text{diagonal}\;\left(\text{Eq}.\left(5\right)\right). \end{array} \end{array}\right.$$

Each nonzero contribution to $p_{x-y}\left(k\right)$ has a weight of $1/{\tilde{N}}_{g}$, so the Contrast feature becomes:

(9) $$\begin{array}{l} f_{2}=\sum_{k=0}^{N_{g}-1}k^{2}p_{x-y}\left(k\right)=\frac{1}{{\tilde{N}}_{g}}\left[{\left(\left|d\right|\nabla \right)}^{2}m_{1}+{\left(-m_{1}\nabla \right)}^{2}m_{2}\right]\\ \;\;\;=\nabla^{2}\left|d\right|\left(\left\lfloor \frac{N_{g}-1}{\left|\nabla \right|}\right\rfloor -\left|d\right|+1\right). \end{array}$$

This expression confirms the empirical intuition that, with increasing ∇ or $\left|d\right|$, pixel pairs become more dissimilar, leading to higher contrast. We summarize the scaling law as:

$$f_{2}\left(N_{g},d,\nabla \right)\propto N_{g}^{+1}{\left|d\right|}^{+2}\nabla^{+2}.$$

Real-world results for benign glandular structures, reported in the top-left panel of Fig. 5 in [8], show that the Haralick Contrast feature indeed increases (nonlinearly) with $N_{g}$, as predicted by Eq. (9). The fact that our scaling law suggests an asymptotically linear, rather than nonlinear, increase of $f_{2}$ with $N_{g}$ may be attributed to the simplified linear gradient textures used in our synthetic images.

This scaling law also reveals an inverse relationship between Energy $f_{1}$ and Contrast $f_{2}$:

(10) $$f_{2}=\nabla \left|d\right|\left(\frac{1}{f_{1}}-\left|d\right|\right).$$

Such a relationship is expected: the Energy feature measures image uniformity and decreases with increasing texture complexity, while the Contrast feature increases with local intensity variation, particularly in edge-dense or rough regions. Despite this relationship, the two features are not redundant: $f_{1}$ is independent of the displacement vector d, whereas $f_{2}$ depends explicitly on it.

*3.2.2.2.* Fig. 4b shows numerical results for $f_{2}$ from synthetic images with various one-dimensional linear gradients. For small gradients $\left(\nabla =\left\{1,3\right\}\right)$, $f_{2}$ varies nearly linearly with $\left|d\right|$, consistent with Eq. (9) in the limit ${\tilde{N}}_{g}\gg \left|d\right|$. In this case, $f_{2}\propto \nabla \left|d\right|N_{g}$. As ∇ increases and ${\tilde{N}}_{g}$ decreases, the quadratic $\left|d\right|$ term becomes more pronounced. The scaling law also predicts a maximum $f_{2,\max}={\left(\nabla {\tilde{N}}_{g}/2\right)}^{2}$ for $\left|d\right|={\tilde{N}}_{g}/2$, which aligns with the trends observed in the figure. At fixed $\left|d\right|$, $f_{2}$ grows nonlinearly with ∇, due to the discreteness introduced by the floor function in ${\tilde{N}}_{g}$.

*3.2.2.3.* For the Haralick Contrast feature $f_{2}$, Löfstedt et al. proposed the empirical normalization $f_{2,\text{Lofstedt}}=f_{2}/N_{g}^{2}$. However, our analytical result (Eq. (9)) shows that $f_{2}\propto N_{g}$, a result confirmed by simulations (Fig. 4b). Thus, dividing the Haralick Contrast feature by $N_{g}^{2}$ constitutes an overcorrection; our scaling law in Eq. (9) predicts an inverse dependence on $N_{g}$. Indeed, the upper-right panel of Fig. 5 from [8] shows that $f_{2,\text{Lofstedt}}$ decreases with $N_{g}$, rather than remaining independent of it as expected. Therefore, the correct normalization of the Haralick Contrast feature is:

(11) $${\tilde{f}}_{2}=\frac{f_{2}}{N_{g}}=\frac{1}{N_{g}}\sum_{k=0}^{N_{g}-1}k^{2}p_{x-y}\left(k\right).$$

#### 3.2.3. Scaling law for correlation (COR)

**Correlation (COR)**, defined in Eq. (19), requires the marginal distributions $p_{x}$ and $p_{y}$, along with their means $\mu_{x}$, $\mu_{y}$, and standard deviations $\sigma_{x}$, $\sigma_{y}$. The Haralick Correlation feature measures the linear dependency or structural similarity between the gray levels of pixel pairs in a specific spatial relationship. It quantifies how predictable a pixel’s value is given the value of its neighbor. A high positive Correlation indicates a strong linear relationship between neighboring pixel values, implying predictable textures or smooth gradients. Low or negative Correlation suggests that pixel intensities are weakly or inversely related, often characteristic of noisy, complex, or nonlinear textures. Haralick Correlation values range from −1 to +1.

*3.2.3.1.* Due to the GLCM symmetries induced by linear gradients (Section 3.1), each reference pixel intensity i=0, ∇, $2\nabla ,\ldots ,\left({\tilde{N}}_{g}-1\right)\nabla$ has a corresponding neighboring intensity $j=i+\nabla \left|d\right|$. As a result, the marginal probability distribution becomes uniform: $p_{x}\left(i\right)=1/{\tilde{N}}_{g}$. The mean and variance are therefore given by:

$$\begin{array}{l} \mu_{x}=\sum_{i=0}^{N_{g}-1}ip_{x}\left(i\right)=\frac{1}{{\tilde{N}}_{g}}\sum_{i=0}^{{\tilde{N}}_{g}-1}i=\frac{\nabla }{2}\left\lfloor \frac{N_{g}-1}{\left|\nabla \right|}\right\rfloor ,\\ \sigma_{x}^{2}=\sum_{i=0}^{N_{g}-1}{\left(i-\mu_{x}\right)}^{2}p_{x}\left(i\right)=\sum_{i=0}^{N_{g}-1}i^{2}p_{x}\left(i\right)-\mu_{x}^{2}\\ \;\;\;=\frac{\nabla^{2}}{12}\left\lfloor \frac{N_{g}-1}{\left|\nabla \right|}\right\rfloor \left(2+\left\lfloor \frac{N_{g}-1}{\left|\nabla \right|}\right\rfloor \right). \end{array}$$

To evaluate the correlation sum:

$$f_{3,\text{sum}}=\sum_{i=0}^{N_{g}-1}\sum_{j=0}^{N_{g}-1}ijp_{d}\left(i,j\right),$$

we use the GLCM symmetries from Eq. (8). The correlation sum simplifies to:

$$f_{3,\text{sum}}=\sum_{i=0}^{N_{g}-1}i\left(i+\left|d\right|\nabla \right)p_{d}\left(i,i+\left|d\right|\nabla \right)+\sum_{i=0}^{N_{g}-1}i\left(i-m_{1}\nabla \right)p_{d}\left(i,i-m_{1}\nabla \right).$$

As a result, the Haralick Correlation feature is given by:

(12) $$f_{3}=\frac{f_{3,\text{sum}}-\mu_{x}^{2}}{\sigma_{x}^{2}}=1-\frac{6\left|d\right|\left({\tilde{N}}_{g}-\left|d\right|\right)}{\left({\tilde{N}}_{g}-1\right)\left({\tilde{N}}_{g}+1\right)}.$$

Based on Eq. (12), $1-f_{3}$ scales quadratically with $\left|d\right|$, similar to $f_{2}$, but exhibits an inverse relationship with ${\tilde{N}}_{g}$:

$$1-f_{3}\propto \nabla {\left|d\right|}^{2}N_{g}^{-1}.$$

Therefore, our analytically derived scaling law for $f_{3}$ is:

$$f_{3}\left(N_{g},d,\nabla \right)\propto 1-\nabla {\left|d\right|}^{2}N_{g}^{-1}.$$

Real-world results for benign glandular structures, reported in the middle-left panel of Fig. 5 in [8], show that the Haralick Correlation feature saturates at (or close to) 1, regardless of the values $N_{g}\in \left\{32,64,96,128,160,192,224,256\right\}$. This is consistent with our scaling laws, which predict $1-f_{3}\propto N_{g}^{-1}$. As $N_{g}$ increases, the Haralick Correlation feature asymptotically approaches $f_{3}\to 1$.

*3.2.3.2.* The numerically computed $f_{3}$ values in Fig. 4c confirm the scaling behavior predicted by Eq. (12). The upper limit of $f_{3}$ approaches 1, as expected. $f_{3}$ shares the same $\left|d\right|\left({\tilde{N}}_{g}-\left|d\right|\right)$ structure as $f_{2}$, which explains their visual similarity. The negative slope arises from the second term in Eq. (12). For large $N_{g}$ and small ∇, $f_{3}\propto \left|d\right|\nabla /N_{g}$, indicating linear dependence on ∇.

*3.2.3.3.* The Haralick Correlation feature used in [8] is identical to the original Haralick definition, i.e., $f_{3,\text{Löfstedt}}=f_{3}$. Our scaling law from Eq. (12) suggests that, as $N_{g}$ increases, the Correlation asymptotically approaches $f_{3}\to 1$. Therefore, our normalized Haralick Correlation co-incides with Haralick’s original definition (and with the normalization used in [8]):

(13) $${\tilde{f}}_{3}=\frac{\sum_{i=0}^{N_{g}-1}\sum_{j=0}^{N_{g}-1}ijp\left(i,j\right)-\mu_{x}\mu_{y}}{\sigma_{x}\sigma_{y}}.$$

#### 3.2.4. Scaling law for homogeneity or Inverse Difference Moment (IDM)

**Homogeneity**, also known as the **Inverse Difference Moment (IDM)**, is defined in Eq. (23). This feature measures the closeness of the distribution of elements in the GLCM to its principal diagonal, where i=j. It quantifies the similarity between neighboring pixels in intensity. High Haralick Homogeneity indicates small intensity differences between adjacent pixels, corresponding to smooth and uniform regions. Low Haralick Homogeneity reflects larger variations in neighboring pixel intensities, characteristic of coarse or noisy textures.

*3.2.4.1.* Given the GLCM symmetries, the summation for this feature simplifies as follows:

(14) $$\begin{array}{l} f_{5}=\sum_{i,j}\frac{1}{1+\left|i-j\right|}p_{d}\left(i,j\right)=\sum_{i}\frac{1}{1+\left|d\right|\nabla }p_{d}\left(i,i+\left|d\right|\nabla \right)\\ \;\;\;\;\;+\sum_{i}\frac{1}{1+m_{1}\nabla }p_{d}\left(i,i-m_{1}\nabla \right)\\ \;\;=\frac{1}{{\tilde{N}}_{g}}\left(\frac{m_{1}}{1+\left|d\right|\nabla }+\frac{\left|d\right|}{1+\left(1+m_{1}\nabla \right)}\right)\\ \;\;=\frac{1}{{\tilde{N}}_{g}}\left(\frac{{\tilde{N}}_{g}-\left|d\right|}{1+\left|d\right|\nabla }+\frac{\left|d\right|}{1+\left(1+{\tilde{N}}_{g}-\left|d\right|\nabla \right)}\right). \end{array}$$

The IDM feature exhibits a complex scaling behavior with respect to ${\tilde{N}}_{g}$. In Eq. (14), the first term becomes asymptotically independent of ${\tilde{N}}_{g}$, while the second term scales approximately as the inverse square of ${\tilde{N}}_{g}$. The quadratic dependence of $f_{5}$ on $N_{g}$ is also shown in the lower-left panel of Fig. 5 from [8], consistent with our scaling law in Eq. (14). As a result, $f_{5}$ is a strong candidate among Haralick features due to its reduced sensitivity to gray-level quantization when appropriately normalized, thereby supporting reproducible texture classification [6,7]. Furthermore, in the limit of large ${\tilde{N}}_{g}$, $f_{5}$ becomes independent of the displacement vector d and inversely proportional to the image gradient ∇. These properties make IDM a robust and quantization-invariant descriptor for characterizing image gradients.

3.2.4.2. Fig. 4d shows $f_{5}$ values numerically computed from synthetic images, which match the predictions of Eq. (14). For large ${\tilde{N}}_{g}$, the first term in Eq. (14) dominates and scales as $1/\left(\left|d\right|\nabla \right)$, producing the hyperbolic shape seen in Fig. 4d. As ∇ increases, ${\tilde{N}}_{g}$ decreases, and the second term in Eq. (14) becomes more significant. For large $\left|d\right|$, the second term becomes independent of $\left|d\right|$ and asymptotically approaches $1/\left(\nabla {\tilde{N}}_{g}\right)$, consistent with the numerical results.

*3.2.4.3.* Real-world benign glandular structure results reported in the lower-right panel of Fig. 5 in [8] show that their normalized Haralick Homogeneity feature remains constant regardless of the values of $N_{g}\in \left\{32,64,96,128,160,192,224,256\right\}$. This is because they replaced the term $1/\left(1+{\left(i-j\right)}^{2}\right)$ in Haralick’s original definition (Eq. (23)) with $1/\left(1+{\left(i/N_{g}-j/N_{g}\right)}^{2}\right)$. As a result, the modified Haralick Homogeneity reduces to:

$$f_{5,\text{Lofstedt}}=\sum_{i=0}^{N_{g}-1}\sum_{j=0}^{N_{g}-1}p\left(i,j\right)=1.$$

However, our scaling law given by Eq. (14) shows that the Homogeneity feature $f_{5}$ exhibits asymptotic invariance to $N_{g}$, as the first term is independent of $N_{g}$ and the second term asymptotically vanishes as $N_{g}$ increases. Therefore, no normalization factor is required:

$${\tilde{f}}_{5}=f_{5}.$$

As a side note, the mean $\mu_{x}\propto N_{g}$ and variance $\sigma_{x}^{2}\propto N_{g}^{2}$ used in the calculation of $f_{3}$ are not themselves invariant, as shown in Eq. (22). Thus, proper normalization of these intermediate quantities should use $\mu_{x}/N_{g}$ and $\sigma_{x}^{2}/N_{g}^{2}$, which was correctly implemented in [8].

To conclude, Löfstedt et al. [8] and Clausi [44], among others, proposed empirical normalizations of Haralick features, which are sometimes appropriate. One drawback of empirical normalization is that it may reduce sensitivity to subtle intensity differences due to over-normalization. In contrast, our analytic framework yields closed-form normalization strategies — such as scaling Haralick Contrast by $N_{g}$ instead of $N_{g}^{2}$ — thereby preserving interpretability. These insights confirm the predictive accuracy of our analytical approach and underscore the importance of robust normalization. In the next section, we derive normalization factors that render Haralick features invariant — or asymptotically insensitive — to variations in gray-level quantization and displacement magnitude.

##### Summary of scaling laws.

The influence of ∇ and $\left|d\right|$ on Haralick features extends to real-world domains, including radiomics, remote sensing, and biomedical image classification. As illustrated in Fig. 4, increasing ∇ reduces the spread of co-occurrences in the GLCM, thereby increasing $f_{1}$ and decreasing $f_{5}$. Conversely, increasing $\left|d\right|$ leads to higher $f_{2}$ and lower $f_{3}$. These scaling laws are consistent with prior empirical observations of Haralick features [6,7,41,44,47].

In radiomics, where textural heterogeneity serves as a biomarker for malignancy [22,43], uncorrected scaling effects can lead to non-reproducible results. Our analytical framework provides a principled approach to standardizing texture descriptors, aligning with both statistical theory and the perceptual insights originally proposed by Haralick et al. [16,17]. All scaling laws derived analytically were validated against synthetically generated images containing one-dimensional linear gradients.

The scaling laws also reveal a functional relationship between Energy $\left(f_{1}\right)$ and Contrast $\left(f_{2}\right)$ for linear gradients (see Eq. (10)). Including both features in a predictive model may introduce multicollinearity, reinforcing the importance of feature reduction techniques grounded in analytical theory rather than empirical heuristics alone.

Understanding feature sensitivity to $N_{g}$ and $\left|d\right|$ enables more informed feature selection. For example, in high-resolution imaging scenarios, Homogeneity $\left(f_{5}\right)$ remains stable, while Energy $\left(f_{1}\right)$ deteriorates with increasing gray-level quantization. This highlights the importance of scaling-aware feature engineering, particularly in radiomics applications.

From a practical perspective, analytic scaling laws offer significant computational advantages. Traditional Haralick feature computation requires $𝒪\left(N_{g}^{2}\right)$ operations per GLCM, creating bottlenecks in large-scale pipelines. In contrast, our closed-form scaling laws enable constant-time feature estimation once ∇ and $\left|d\right|$ are known, significantly improving throughput in batch-processing environments such as radiomics or remote sensing. Furthermore, these formulas can be seamlessly integrated into pre-processing pipelines, while preserving both speed and reproducibility.

As texture-based features are increasingly integrated into hybrid deep learning models [48,49], the scalability and interpretability of analytically derived descriptors make them ideal for deployment across institutions and heterogeneous imaging platforms.

## 4. Discussion

Due to their practical importance in biomedical image classification, several studies have empirically investigated Haralick feature normalization to improve both portability and classification accuracy. For example, Clausi [44] examined the effect of gray-level quantization on the performance of GLCM-derived features for classifying natural textures. The study utilized Brodatz imagery [50], synthetic aperture radar (SAR) aerial imagery from the Labrador Ice Margin Experiment (LIMEX) [51], and SAR satellite imagery from the North Water (NOW) Polynya project [52]. Clausi analyzed the Inverse Difference Moment (IDM) feature defined in Eq. (23), as well as the Inverse Difference Haralick feature. The study concluded that “to improve the classification ability of these two statistics, the difference between i and j can be normalized by the number of gray levels.” However, as shown in this work, such normalization is unnecessary when using expressions like $\frac{1}{1+\left|i-j\right|}$, which already exhibit asymptotic invariance.

The study also explored how reducing the number of gray levels $N_{g}$ could accelerate GLCM computation and reduce noise sensitivity [44]. However, this comes at the cost of information loss, and “coarser quantization would reduce both classification accuracy and feature space”. An earlier study by Soh and Tsatsoulis [53] similarly concluded that “the information gain in noise-effect reduction does not compensate for the loss of information resulting from quantization”.

Clausi [44] also observed that for the Haralick Contrast feature $f_{2}$, classification performance was largely independent of $N_{g}$ once $N_{g}≳24$. Our results provide a theoretical explanation: this improved performance likely arises from the linear increase of $f_{2}$ with $N_{g}$ (see Eq. (9)), which enhances texture discrimination.

The ASM feature $f_{1}$, referred to as Uniformity (UNI) in [44], was found to exhibit a “strong decrease in classification accuracy with increasing” $N_{g}$. The study remarked that “a decrease in classification accuracy with increasing $N_{g}$ was not expected.” Our scaling law for ASM in Eq. (6) shows that $f_{1}$ is inversely proportional to $N_{g}$, which explains why increasing $N_{g}$ diminishes the discriminative power of $f_{1}$, consistent with Clausi’s observations.

Regarding IDM $\left(f_{5}\right)$, referred to as the Inverse Difference (INV) feature in [44], the study reported “decreasing classification accuracy with increasing” $N_{g}$. However, Fig. 3 in [44] shows that $f_{5}$ maintains high accuracy and is nearly invariant to $N_{g}$ across the Brodatz, LIMEX, and NOW datasets. These findings align with our scaling law in Eq. (14), where the first term is independent of $N_{g}$ and the second term vanishes as $N_{g}$ increases. This may explain the higher $f_{5}$ values observed at small $N_{g}<100$, and the plateau reached at larger $N_{g}$, as reported by Clausi.

Clausi [44] further noted that both the Contrast feature $\left(f_{2}\right)$ and the Correlation feature $\left(f_{3}\right)$ produced stable classification results when $N_{g}>24$. Our analytical scaling laws (Eq. (9) for $f_{2}$ and Eq. (12) for $f_{3}$) show that both features increase with $N_{g}$, helping to explain their robust performance in classification tasks under finer quantization.

These findings highlight the importance of selecting Haralick features with stable scaling behavior when designing classifiers for images with varying resolution or quantization. Features with invariant scaling, such as $f_{5}$ and $f_{3}$, offer improved generalization and can be reliably used across heterogeneous datasets. In contrast, Haralick features that are sensitive to the quantization scheme, such as $f_{1}$, benefit from normalization factors such as those derived in this study to ensure consistency and reproducibility.

## 5. Conclusion

Image gradients are fundamental building blocks in image processing. In this study, we used linear gray-level gradients to investigate the symmetries induced in the gray-level co-occurrence matrix (GLCM), and leveraged these symmetries to simplify the analytical derivation of scaling laws for selected Haralick features. While a higher bit depth (i.e., a larger number of gray levels $N_{g}$) provides greater information content, it also demands increased computational resources.

Among various applications, image gradients are employed in edge detection, deepfake detection, and pattern classification. We found that linear gradients produce nonzero GLCM entries located along diagonals offset from the principal diagonal, with the offset determined by the product of the gradient intensity ∇ and the magnitude of the displacement vector $\left|d\right|$. The gradient intensity ∇ (in gray levels per pixel) determines the number of nonzero entries in the GLCM: ${\tilde{N}}_{g}=\left\lfloor \frac{N_{g}-1}{\left|\nabla \right|}\right\rfloor +1$. The offset from the principal diagonal is given by $k=\left|i-j\right|$, where i is the gray level at pixel $\left(x_{i},y_{i}\right)$, and j is the gray level at pixel $\left(x_{j}=x_{i}+\Delta x,y_{j}=y_{i}+\Delta y\right)$.

Due to the GLCM symmetry induced by linear gradients, we derived analytic scaling laws for four widely used Haralick features. The derivation procedure can be extended to additional features. We observed strong agreement between the analytically derived scaling laws and numerically computed Haralick features using MATLAB’s built-in functions.

Although the scaling laws were derived under the assumption of linear gradients, they proved effective for identifying normalization factors that render Haralick features invariant — or at least less sensitive — to variations in $N_{g}$. These scaling laws provide a principled and consistent framework for estimating normalization factors across diverse imaging contexts. Our normalization strategies were found to be effective even for heterogeneous datasets, including Brodatz textures, SAR data from LIMEX and the NOW Polynya project [44], and benign glandular structures from the gland dataset [8]. A likely explanation is that linear gradients, as elemental components of texture, encode intrinsic statistical properties common across many image types.

The detailed analysis of linear gradients, their associated GLCM symmetries, and the resulting Haralick scaling laws aligns with structural patterns observed across numerous datasets. Notably, these scaling laws can be inverted to estimate gray-level gradients from observed Haralick values. However, this inversion is non-unique due to the presence of the floor function in the expression ${\tilde{N}}_{g}=\left\lfloor \frac{N_{g}-1}{\left|\nabla \right|}\right\rfloor$.

A promising direction for future research is to conduct sensitivity analyses of analytically derived features to identify optimal quantization levels and displacement vector ranges that maximize discriminative power. Classifiers built on Haralick features should prioritize those with the highest task-relevant sensitivity to improve accuracy and generalizability.

## Figures and Tables

**Table 1:** Normalization factors for Haralick features based on scaling laws.

Feature	Scaling law	Normalization
$f_{1}\left(\text{ASM}\right)$	^ $\propto \nabla^{+1}N_{g}^{-1}$ ^	${\tilde{f}}_{1}=f_{1}N_{g}$
$f_{2}\left(\text{Contrast}\right)$	$\propto \nabla^{+2}{\left|d\right|}^{+2}N^{+1}$	${\tilde{f}}_{2}=f_{2}/N_{g}$
$f_{3}\left(\text{Correlation}\right)$	$1-\nabla {\left|d\right|}^{2}N_{g}^{-1}$	${\tilde{f}}_{3}=f_{3}$
$f_{5}\left(\text{IDM}\right)$	$\propto \nabla^{-1}\left(\text{asymptotically}\right)$	${\tilde{f}}_{5}=f_{5}$

## Appendix A. Selected Haralick features

While there is no universally accepted notation for Haralick features, we follow the original nomenclature and labeling from [16,17]. Throughout this section, $p\left(i,j\right)$ denotes the normalized gray-level co-occurrence matrix (GLCM) for a displacement vector $d=\left(\Delta x,\Delta y\right)$, and $N_{g}$ is the number of gray levels in the quantized image.

**Angular Second Moment (ASM)** or **Energy** measures image homogeneity and is defined as [16,17]:

(16)
$$f_{1}=\sum_{i=0}^{N_{g}-1}\sum_{j=0}^{N_{g}-1}p{\left(i,j\right)}^{2}.$$

When neighboring pixel intensities are similar, the ASM value is high.

**Contrast (CON)** measures the local variation in gray levels and is defined as [16,17]:

(17)
$$f_{2}=\sum_{k=0}^{N_{g}-1}k^{2}p_{x-y}\left(k\right),$$

where the marginal distribution is [16,17]:

(18)
$$p_{x-y}\left(k\right)=\sum_{i=0}^{N_{g}-1}\sum_{j=0}^{N_{g}-1}\delta_{\left|i-j\right|,k}p\left(i,j\right),$$

and $\delta_{m,n}$ is the Kronecker delta. The function $p_{x-y}\left(k\right)$ aggregates all GLCM entries having a fixed absolute gray-level difference $k=\left|i-j\right|$ between reference pixel i and neighboring pixel j.

For example, $p_{x-y}\left(0\right)$ corresponds to the sum of the GLCM diagonal entries $\left(i=j\right)$, and $p_{x-y}\left(1\right)$ corresponds to the sum of entries along lines where $\left|i-j\right|=1$ (see Figs. 2 and 3). When i=j, the image is locally uniform, contributing k=0 to $f_{2}$, which is minimal. Larger differences between i and j increase $f_{2}$, since $k^{2}$ grows quadratically. If the GLCM is populated only along its principal diagonal, then $f_{2}=0$.

**Correlation (COR)** measures the linear dependency between the gray levels of neighboring pixels and is defined as [16,17]:

(19)
$$f_{3}=\frac{\sum_{i=0}^{N_{g}-1}\sum_{j=0}^{N_{g}-1}ijp\left(i,j\right)-\mu_{x}\mu_{y}}{\sigma_{x}\sigma_{y}},$$

where the marginal distributions are:

(20)
$$p_{x}\left(i\right)=\sum_{j=0}^{N_{g}-1}p\left(i,j\right),p_{y}\left(j\right)=\sum_{i=0}^{N_{g}-1}p\left(i,j\right).$$

The means and variances are defined as:

(21)
$$\mu_{x}=\sum_{i=0}^{N_{g}-1}ip_{x}\left(i\right),\;\;\;\;\;\;\;\;\;\;\;\;\;\;\;\;\;\;\;\;\;\;\;\;\;\;\;\mu_{y}=\sum_{j=0}^{N_{g}-1}jp_{y}\left(j\right),$$

(22)
$$\sigma_{x}^{2}=\sum_{i=0}^{N_{g}-1}{\left(i-\mu_{x}\right)}^{2}p_{x}\left(i\right),\;\;\;\;\;\;\;\;\;\;\;\;\;\;\;\;\;\;\;\sigma_{y}^{2}=\sum_{j=0}^{N_{g}-1}{\left(i-\mu_{y}\right)}^{2}p_{y}\left(j\right).$$

The correlation value typically ranges between −1 and 1. A value near 0 indicates no spatial correlation, while values close to ±1 indicate strong positive or negative linear relationships between neighboring gray levels.

**Inverse Difference Moment (IDM)** measures local homogeneity and is defined as [16,17]:

(23)
$$f_{5}=\sum_{i=0}^{N_{g}-1}\sum_{j=0}^{N_{g}-1}\frac{1}{1+{\left(i-j\right)}^{2}}p\left(i,j\right).$$

The weights $1/\left(1+{\left(i-j\right)}^{2}\right)$ decay quadratically with increasing $\left|i-j\right|$, emphasizing values near the principal diagonal of the GLCM. IDM attains high values in homogeneous regions.

A commonly used variant, known as **Homogeneity**, is defined as [16,17]:

(24)
$$\sum_{i=0}^{N_{g}-1}\sum_{j=0}^{N_{g}-1}\frac{1}{1+\left|i-j\right|}p\left(i,j\right).$$

This version decays linearly, rather than quadratically, but still emphasizes entries close to the diagonal of the GLCM.

## Appendix B. Implementation considerations for Haralick features

The third column in Table 1 presents the relationship between our proposed normalized features, based on analytically derived scaling $\tilde{f}$, and the existing implementations of the unnormalized Haralick features f. Implementations of unnormalized Haralick features f are available in various programming languages. For example, in MATLAB, the GLCM can be generated from an image using the command:

GLCMS = graycomatrix(image, ‘Offset’, offsets); followed by extracting the unnormalized Haralick features from the GLCM with the command:

features = graycoprops(GLCMS, ‘Contrast’, ‘Correlation’, ‘Energy’, ‘Homogeneity’);

A more complete, custom-designed Haralick feature function is also available from MATLAB at https://www.mathworks.com/matlabcentral/fileexchange/58769-haralicktexturefeatures. This function implements all 14 Haralick features. In Python, there are also many available custom-designed unnormalized feature implementations. For example, https://www.geeksforgeeks.org/python/mahotas-haralick-features/ demonstrates a few library calls:

import mahotas as mh

import mahotas.features

import numpy as np

haralick_features = mh.features.haralick(gray_image)

Once the unnormalized original Haralick features are determined, one can use the summary Table 1 to find their normalized equivalents. For example, the normalized Haralick feature $\tilde{f}$ can be obtained by multiplying the unnormalized value $f_{1}$, as obtained from MATLAB or Python, by the number of gray levels $N_{g}$.

There is no need to modify the existing optimized algorithms for unnormalized Haralick feature calculations. After computing $f_{1}$ in MATLAB, Python, or any other language that implements such a function, it suffices to multiply $f_{1}$ by $N_{g}$ to obtain the normalized $\tilde{f}$.

## Glossary.

### Abbreviations

b: bit depth, i.e., a positive integer that specifies how many bits are used to encode light intensities in an image. Black-and-white images use one-bit encoding, where 0 represents black and 1 represents white.
$d=\left(\Delta x=x_{j}-x_{i},\Delta y=y_{j}-y_{i}\right)$: displacement vector, i.e., the offset between the reference pixel (with intensity i at coordinates $\left(x_{i},y_{i}\right)$) and the target pixel (with intensity j at coordinates $\left(x_{j},y_{j}\right)$).
$\left|d\right|=\sqrt{\Delta x^{2}+\Delta y^{2}}$: magnitude of the displacement vector.
$f_{1}$: Haralick’s Angular Second Moment (ASM), or Energy, which measures image uniformity. Its normalized version is denoted ${\tilde{\text{f}}}_{\text{1}}$.
$f_{2}$: Haralick’s Contrast, which measures local intensity variation based on the differences between neighboring pixel pairs. Its normalized version is ${\tilde{\text{f}}}_{\text{2}}$.
$f_{3}$: Haralick’s Correlation, which quantifies the linear dependency or structural similarity between the gray levels of pixel pairs. Its normalized version is ${\tilde{\text{f}}}_{\text{3}}$.
$f_{5}$: Haralick’s Homogeneity (or Inverse Difference Moment), which measures the closeness of the GLCM distribution to its principal diagonal, where i=j. Its normalized version is ${\tilde{\text{f}}}_{\text{5}}$.
$m_{1}$ and $m_{2}$: numbers of GLCM entries parallel to and above, respectively below, the principal diagonal, associated with an image gradient of intensity ∇.
$\mu_{x}$ and $\mu_{y}$: means of the marginal distributions $p_{x}\left(i\right)$ and $p_{y}\left(j\right)$, respectively.
∇: image gradient, i.e., the gray-level intensity difference $\left|i-j\right|$ between a reference pixel and a target pixel separated by a displacement vector d.
$N_{g}$: number of gray levels in the image, i.e., a positive integer determined by the bit depth: $N_{g}=2^{b}$. For a black-and-white image, $N_{g}=2^{1}=2$, where 0 represents black and 1 represents white.
${\tilde{N}}_{g}$: number of nonzero entries in the GLCM.
$P_{d}\left(i,j\right)$: Gray-Level Co-occurrence Matrix (GLCM), an $N_{g}\times N_{g}$ matrix that counts the number of pixel pairs with reference intensity i and target intensity j, separated by a displacement vector $d=\left(\Delta x,\Delta y\right)$. The normalized GLCM is denoted ${\text{P}}_{\text{d}}\left(\text{i},\text{j}\right)$. The GLCM is typically sparse, with ${\tilde{N}}_{g}$ nonzero entries.
$p_{x}\left(i\right)$: marginal distribution representing the probability of encountering reference intensity i in the image, regardless of the target intensity j.
$p_{y}\left(j\right)$: marginal distribution representing the probability of encountering target intensity j, regardless of the reference intensity i.
$p_{x-y}\left(k\right)$: marginal distribution of GLCM entries with a fixed absolute gray-level difference $k=\left|i-j\right|$ between the reference and neighboring pixels.
$p_{x+y}\left(k\right)$: marginal distribution of GLCM entries with a fixed gray-level sum k=i+j between the reference and neighboring pixels.
$R_{x}$ and $R_{y}$: total numbers of horizontal and vertical pixel pairs, respectively, at unit distance. These serve as normalization factors for $P_{d}\left(i,j\right)$.
$\sigma_{x}^{2}$ and $\sigma_{y}^{2}$: variances of the marginal distributions $p_{x}\left(i\right)$ and $p_{y}\left(j\right)$, respectively.
